# Can life coaching improve health outcomes? – A systematic review of intervention studies

**DOI:** 10.1186/1472-6963-13-428

**Published:** 2013-10-22

**Authors:** Jette Ammentorp, Lisbeth Uhrenfeldt, Flemming Angel, Martin Ehrensvärd, Ebbe B Carlsen, Poul-Erik Kofoed

**Affiliations:** 1 Health Services Research Unit, Lillebaelt Hospital/IRS University of Southern Denmark, Kabbeltoft, Vejle, Denmark; 2 Horsens Hospital Research Unit/ Department of Public Health, Aarhus University, Sundvej 30, Horsens, Denmark; 3 Faculty of Theology, University of Copenhagen, Købmagergade 44-46, Copenhagen, Denmark; 4 Department of Paediatrics, Lillebaelt Hospital/IRS University of Southern Denmark, Skovvangen 2-8, Kolding, Denmark

**Keywords:** Life coaching, Health coaching, Intervention, Review, Patient outcomes, Communication

## Abstract

**Background:**

In recent years, coaching has received special attention as a method to improve healthy lifestyle behaviours. The fact that coaching has found its way into healthcare and may provide new ways of engaging the patients and making them accountable for their health, justifies the need for an overview of the evidence regarding coaching interventions used in patient care, the effect of the interventions, and the quality of the studies published. However, in order to provide a clear definition of the coaching interventions selected for this review, we have found it necessary to distinguish between health coaching and life coaching. In this review, we will only focus on the latter method and on that basis assess the health related outcomes of life coaching.

**Methods:**

Intervention studies using quantitative or qualitative methods to evaluate the outcome of the life coach interventions were identified through systematic literature searches in PubMed, Embase, Psycinfo, and CINAHL. The quality of the methodology was independently assessed by three of the authors using a criteria list.

**Results:**

A total of 4359 citations were identified in the electronic search and five studies were included; two of them were randomized controlled trials and met all quality criteria. The two studies investigating objective health outcomes (HbA1c) showed mixed but promising results, especially concerning the patient group that usually does not benefit from intensified interventions.

**Conclusion:**

Because of the very limited number of solid studies, this review can only present tendencies for patient outcomes and a preliminary description of an effective life coaching intervention.

The coaching method used in these studies aims to improve self-efficacy and self-empowerment. This may explain why the studies including disadvantaged patients showed the most convincing results. The findings also indicate that some patients benefit from being met with an alternative approach and a different type of communication than they are used to from health care personnel.

In order to get a closer look at what is in the ‘black box’, we suggest that the description and categorisation of the coaching methods are described more comprehensively, and that research into this area is supplemented by a more qualitative approach.

## Background

Coaching is a method that has proven useful in enhancing personal insight and in shaping and reinforcing desired behaviour within many different contexts [1]. In recent years, coaching has received special attention as a method to improve healthy lifestyle behaviours [2].

It is well-known that having the insight and resources needed to make choices that foster a healthy lifestyle is essential for patients. A healthy lifestyle is important for optimal outcome in patient care and to prevent many of the lifestyle diseases that are dramatically increasing in frequency during these years [3,4]. In a review about strategies for improving the outcome of the treatment of diabetic patients, coaching has been suggested as a supplemental method [5,6]. And the increasing number of papers about coaching interventions shows that coaching is now being used in a wide range of chronic patients.

We know that it is a research area hampered by insufficient design and lack of conceptual clarity [2]. However, the fact that coaching has found its way into healthcare and may provide new ways of engaging the patients and making them accountable for their health [1,7], justifies the need for an overview of the evidence regarding coaching interventions used in patient care, the effect of the interventions, and the quality of the studies published.

Coaching has developed from a wide range of disciplines and is based on broad academic knowledge including cognitive and behavioral psychology, social science, positive psychology, and organizational change and development. There is no precise definition of coaching, but it has been described as a method to “unlocking a person’s potential to maximise their own performance” [8], to encourage patients to acknowledge their creativity and to find their own unique solutions by focusing on the present and being goal-oriented [9-11]. There are several aspects common to nearly all forms of coaching such as the core assumption that people have an innate capacity to grow and develop, as well as a focus on constructing solutions and goal attainment processes rather than just analyzing the problems. Furthermore, the coaching process is viewed as a systematic process and is typically directed at fostering the ongoing self-directed learning and personal growth of the client [8].

However, in order to provide a clear definition of the coaching interventions selected for this review and minimize the risk of mixing the interventions with other cognitive and behavioural methods, we have found it necessary to distinguish between health coaching and life coaching.

Health coaching has been described as “a practice of health education and health promotion within a coaching context, to enhance the well-being of individuals, and to facilitate the achievement of their health-related goals” [12]. The term “health coaching” has emerged from the motivational interviewing concept (MI) originated by William Miller [13] and some of the studies described as health coaching are primarily based on the MI strategy [2] while most of the studies investigating the effect of MI are not described as coaching interventions [14,15]. In order to avoid methodological confusion, we included only life coaching studies in this review.

Health coaching differs from life coaching by focusing on specific health-related topics and goals, while in life coaching the clients may come to the sessions with whatever issues they would like to address. The aim of life coaching is sustained cognitive, emotional, and behavioural changes that facilitate goal attainment and performance enhancement [16]. The life coach meets the clients with the approach that our attitudes and judgements determine our feelings, decisions, and behaviour. Therefore, the coach will help the client unravel distortions in thinking and enable them to learn alternative ways to approach the world in order to improve decision making and help them achieve their goals [16].

Life coaching is based on the assumption that the issues most important to the client are self-identified and self-prioritized, and therefore, it is the clients that choose the topic, the action, and the results that they want to achieve [17,18]. Furthermore, life coaching is defined as focusing on the person’s whole life and by focusing on wellness rather than pathology [17].

In accordance with the approach in patient-centred care [19], life coaching is based on the needs, values, and priorities of the patients. Therefore, we find it highly relevant to identify studies investigating the effect of life coaching on patient outcome.

In an annotated bibliography from 2009 [20] investigating life coach studies on health-related outcomes (blood pressure, weight, quality of life, physical activity, depression, and emotional distress), a total of 72 studies were described and annotated. The study population included healthy participants with or without lifestyle problems as well as patients with a diagnosis. The author concluded that a number of the studies showed life coaching to be a valuable intervention within a wide range of health-related issues. However, the main limitation in most studies was the lack of an operational definition which made it difficult to understand what was intended by the term ‘coaching’ and what distinguished coaching from education, instruction, and motivating counselling [20].

On that basis the major research question in this review is as follows:

– Assess the health-related outcomes of life coaching interventions conducted with patients in the form of individual telephone coaching, individual face-to-face coaching, group coaching, or coaching that combines some or all of these methods.

## Methods

A systematic literature search was conducted in order to examine published studies describing coaching interventions designed to improve health behaviour, patient self-care, and/or health outcomes.

### Criteria for considering studies for this review

#### Types of studies

The studies eligible for inclusion were intervention studies using quantitative or qualitative methods to evaluate the outcome of the coaching or a combination of the methods.

#### Types of participants

Adult somatic patients > 13 years identified in healthcare.

#### Types of intervention

The coaching intervention eligible for inclusion only included methods that were in accordance with the description of life coaching [12,17].

It included studies in which the coaching was as follows:

•based on the agenda of the patient and reflecting the present wishes and needs of the patient. The dialogue was holistic, individualized, and non-programmatic.

•conducted by professional coaches or healthcare professionals with special training in coaching

•conducted as face-to-face, telephone, or internet coaching or a combination of these methods; and

•individual or group sessions or a combination of both methods.

### Criteria for excluding studies

As a consequence of the description of the coaching method, interventions characterized by an externally defined and fixed agenda, such as learning programs and health promotion programs, were excluded, as were coaching interventions that were part of a program and not separately evaluated.

Coaching interventions targeting parents of paediatric patients were also excluded.

### Search strategy

The literature search was conducted between December 2011 and January 2013 using PubMed, Embase, Psycinfo, and CINAHL. There was no time limit set for the search and the keywords used were search terms and synonyms formulated on the basis of the population (patients), the intervention (coaching), and the outcomes (health behaviour, self-care, and health outcomes). Relevant published studies were reviewed for additional keywords and synonyms. The search strategy was established by grouping the individual free text and MESH terms into categories and by combining those components. The research strategy was adapted to each database. To minimize the risk of overlooking relevant literature, a manual search of key journals and of reference lists of included articles was conducted. No language restriction was used.

The search was complemented by determining which terms were used for coaching in the following countries and languages: Germany (‘coaching’); Italy (‘coaching’); France (‘coaching’ or ‘accompagnement’); Spain (‘coaching’); Russia ; and Israel . However, including the extra terms in the search did not yield further literature meeting the inclusion criteria.

### Data collection and analysis

#### Selection of studies

One reviewer (FA) independently applied inclusion criteria to all of the titles and abstracts identified by the electronic searches. During the process, uncertainties about the findings and the search strategy were discussed with JA and EC.

The articles that were evaluated to be potentially relevant were independently assessed by two reviewers (LU and JA) against the inclusion and exclusion criteria. Any disagreements were resolved during face-to-face meetings with all authors and discussed with PK and ME.

#### Data extraction and management

To compare the studies, the following data were extracted from the selected publications: author and year of publication; design; the aim of the study; description of the setting and the population; the intervention; the applied coaching method; and the education of the coaches.

#### Quality assessment

To assess the methodological quality of the included publications, three reviewers (LU, PK, and JA) independently assessed the quality of each eligible study. For that purpose, we used a criteria list inspired by the lists developed by Moja [21], Olsen [2], and Cherafhi-Sohi et al. [22].

Disagreements in the ratings were resolved during face-to-face meetings.

The following 10 criteria were assessed:

1) Random assignment

The reviewers assessed the criterion as ‘done’ if the patients were randomized and the randomisation method was described. If the patients were not randomized, the criterion was assessed as ‘not done’ and it was assessed as ‘not clear’ if there were insufficient details of the allocation method.

2) Use of a control group

If the study used a controlled design, the criterion was assessed as ‘done’ and ‘not done’ if there was no control group.

3) Follow-up

If an outcome measure was obtained for ≥ 80% of the patients, it was assessed as ‘done,’ while it was assessed as ‘not done’ if < 80% of the patients were followed up upon. In cases where it was not specified in the paper, it was assessed as ‘not clear’.

4) Baseline-comparability

The reviewers assessed the criterion as ‘done’ if the authors had performed an analysis of baseline comparability and if the reported findings showed no significant differences. If there were significant baseline differences, it was reported as ‘not done’, and ‘not clear’ if no evidence of baseline comparability was reported.

5) Analysis of data clearly reported

If the analysis of data was sufficiently described, the criterion was assessed as ‘done.’ If the analysis of data was not sufficiently described, it was assessed as ‘not done’ and in cases where it was not specified in the paper, it was assessed as ‘not clear’.

6) Validated outcome measure

The reviewers assessed the criterion as ‘done’ if the outcome measures used were standardized health outcomes or valid and reliable measurement instruments which had been published in peer-reviewed journals. ‘Not done’ was reported if the outcomes measured were not standardized health outcomes or if the measurement instruments were not based on instruments published in peer-reviewed journals. The criterion was assessed as ‘not clear’ if it was not obvious that it was a standardized health outcome, such as HbA1c or blood pressure, or if the measurement instrument was not described.

7) Description of the intervention

The reviewer assessed the criterion as ‘done’ if the intervention was described in detail (format, frequency, length, coaching elements, and coach training) and as ‘not done/not adequate’ if there was no description, or if it was assessed as insufficient.

The maximum criteria score for the publications was 7 points as ‘done’ gave 1 point and ‘not done’ or ‘not clear’ gave 0 points.

### Ethical considerations

As the this study is only based on data collected from the literature; approval from The Danish Scientific Ethical Committee was not required.

## Results

**Figure 1:**
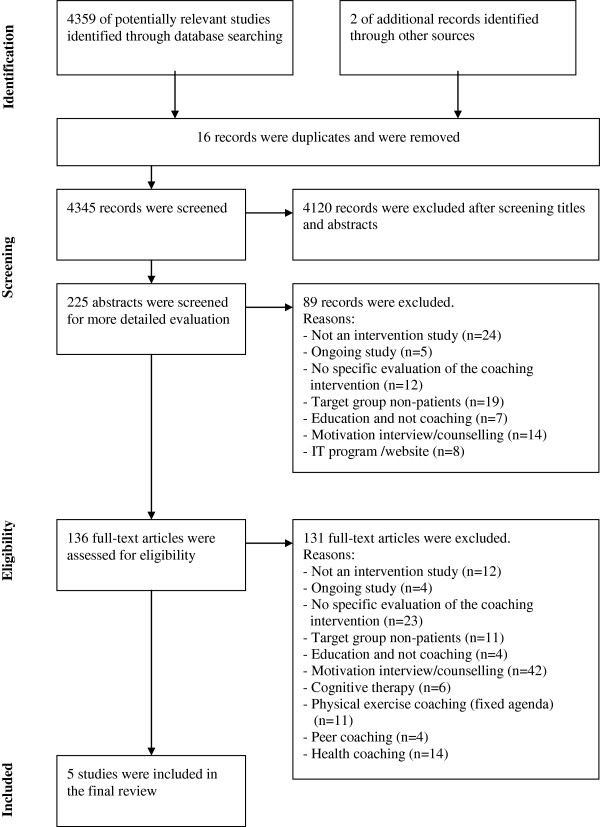
Flowchart of literature search process.

A total of 4359 citations were identified through database searching and 2 were identified through other sources; one of these was one of our own studies. Screening of the titles reduced the number of citations to 225 and after reviewing the abstracts, 136 full papers were retrieved and reviewed. After this, there were 5 studies remaining that met all of the inclusion criteria. Searching by hand did not identify more studies (Figure 1).

Two of the studies were reported across two separate publications each, one of which described the outcome of the randomized trial [23] and the other was a qualitative evaluation [24]. An overview of the studies is presented in Table 1.

**Table 1:** The included studies

**Author**	**Design**	**Aim**	**Population**	**Intervention/duration**	**Coaching method**	**Coach education**	**Patient outcome**
Ammentorp et al. 2013.	Case study using a combination of methods.	To investigate whether or not coaching offered to a group of poorly controlled adolescents with diabetes could improve their self-image, responsibility, and metabolic control.	9 adolescents between 16 and 19 years of age with poorly controlled diabetes for the past 2 years.	The coaching program included: 2 group coaching sessions with all participants and the coaches (4 hours each) 5 individual face-to-face coaching sessions; and 3 telephone coaching sessions. The individual coaching sessions with the personal coach lasted approximately 1½ hours.	The coaching was based on a co-active coaching model. To guide the clients through the process, a Pro-Active Plan for each of the patients was used. The plan included different tools that the adolescent could use as homework, such as: a) “The wheel of life,” by which different aspects of life can be rated, or b) templates for writing down barriers, resources, and the adolescents’ values, goals, milestones, and action plans.	The coaching was conducted by three professional certified coaches with no connection to health service. Before the study started the coaches were introduced to the most common medical terms used in diabetic care.	The mean HbA1c decreased from 11.089% from before coaching to 9.961% (p=0.03) at the end of coaching, but increased slightly 6 months later (10.278% (p=0.047)). The themes generated from the interviews with the adolescent were: “The experience of being met”; “Looking at myself and my diabetes in a new way”; “More self-esteem and more energy”; and “New tools to change routines”.
Galantino et al. 2009	Pre-post intervention study.	To evaluate the immediate and longitudinal impact of 6 wellness coaching sessions for cancer survivors in improving health, fitness, well-being, and overall quality of life.	20 cancer survivors between 35–76 years who ranged between 0.5- 9 years since primary treatment ended.	Telephone coaching that included an initial session lasting 90 minutes and 5 follow up sessions completed over a three-month span lasting 30–40 minutes each.	Wellness coaching (WC) is described as a humanistic, growth-promoting relationship designed for constructive development. It is focusing on what matters most for the patients and on creating a vision and a realistic plan that works within the framework of the patient’s life, enlisting the individual as their own expert. Initially the patients were guided to develop a wellness vision and a behavioural plan. The follow up sessions included reflection of the plan and coaching around any areas of concern.	The coach was an ACSM certified Health Fitness Specialist and certified Wellcoach who was also a breast cancer survivor.	Compared with baseline, the study showed significant improvement in overall quality of life, decreased depression and anxiety, as well as improvement in exercise stage at the completion of the three-month intervention. After 12 months, a slight decline was seen, but did not return to baseline. Non-significant improvements were observed in self-reported physical activity, fruit/vegetable consumption and BMI.
Izumi et al. 2007 Hayashi et al. 2008	a) Randomized controlled trial. Pilot study. b) A qualitative descriptive sub-study.	a) To examine the effect of coaching intervention on psychological adjustment to illness and health-related QOL (HRQOL). b) To analyze and describe subjective evaluations of coaches and intervention subjects on the functions of tele-coaching intervention.	a) 24 patients with spinocerebellar degeneration 20–65 years of age without cognitive impairment or psychiatric disorder.	10 weekly telephone coaching sessions of 15–30 minutes over 3 months.	The coaching intervention was designed to help the patients improve their performance through enhancing psychological adjustment to illness. The process of coaching consisted of six steps: set-up; goal-setting; evaluation of present status; acknowledgement of the gap between the goal and the present status; action-planning to overcome the gap; and follow-up.	The three coaches were experienced physicians (practiced for 19–21 years) trained and supervised by certified coaches. The coaches had experiences with tele-coaching and had been trained in narrative therapy techniques. To control the quality of the coaching, each coach received feedback from patients after sessions 4 and 8 in a survey regarding the attitudes and skills of the coach. Weekly telephone conferences were conducted among the coaches.	a) No statistically significant differences were found between the control and intervention groups. At follow-up, the coaching group had significantly better self-efficacy scores than the control group. b) The tele-coaching enabled patients to tell their own stories in a daily-life setting, encouraged them to experience and adopt fresh points of view, and helped them to start working towards attainable goals without giving up.
Schneider et al. 2011	An intervention study using mixed methods.	To examine how individuals with diabetes perceived life coaching and person-centered planning as an intervention to maintain employment and manage chronic health issues.	108 participants between 18 and 62 years of age with a diagnosis of diabetes, pre-diabetes, or a hemoglobin A1c (HbA1c) > 6.5%; and work at least 40 hours in the preceding month at the federal minimum wage or higher.	Approx. 11 life coach sessions per participant over a 1-year period. Mostly in-person coaching sessions lasting approx. 1 hour. Some telephone coaching sessions, lasting approx. 40 minutes, in addition to a few online sessions lasting 2 hours.	Life coaching was defined as a method by which the client has full control over the topics of the conversation. The main function of the life coach was to assist participants to set and achieve work, health, and personal goals by using SMART (specific, measurable, attainable, realistic, and timed) goals.	Coaches received training in motivational interviewing, the trans-theoretical model (stages of change), and a comprehensive coaching curriculum.	The patients reported high satisfaction with life coaching. The majority of goals were fully or partially achieved (self-reported).
Wolever et al. 2010	Randomized controlled trial.	The purpose of this study was to evaluate the effectiveness of integrative health (IH) coaching on psychosocial factors, behaviour change, and glycemic control in patients with type 2 diabetes.	56 patients at least 18 years of age with a diagnosis of type 2 diabetes for at least 1 year and taking oral diabetes medication for at least 1 year.	14 telephone coaching sessions of 30 minutes (8 weekly sessions, 4 biweekly sessions, and 1 final session a month later).	Integrative health coaching (IH) is defined as a personalized intervention that assists people in identifying their own values and vision of health. Patients were guided in creating a vision of health, and long-term goals aligned with that vision were discussed. The Wheel of Life was used to explore values, establish priorities, and set goals.	Two coaches that were trained in coaching methods and had master’s-level degrees in social work or psychology. Each coach had > 100 hours of experience with coaching diabetes patients.	Compared with baseline, the patients with elevated baseline HbA1c (≥7%) significantly reduced their HbA1c in the intervention group, but not in the control group. Compared with the control group, the coaching group reported that barriers to medication adherence decreased while exercise frequency, stress, and perceived health status increased.

### Participants and setting

Diabetes patients were the focus of the intervention in three of the studies [7,25,26], patients with spinocerebellar degeneration were the focus of the intervention in another study [23,24] and cancer patients in the last study [27].

Three of the included studies were conducted in the United States [25-27] and the remaining studies were conducted in Japan [23,24] and Denmark [7].

### Intervention

All but two studies [26,27] used both face-to-face and telephone coaching, and one of the studies also used group coaching [7]. The number of coaching sessions ranged from 6–14; the coaching sessions were conducted over a period of 3 to 12 months.

#### Aim of the intervention

All studies aimed to investigate the effect of the coaching using quantitative data such as HbA1c and subjective data from questionnaires filled in by the patients. Three of the studies also included a qualitative evaluation of the intervention [7,24,25].

#### Coaching method

The coaching methods were described in all of the studies and were in agreement with the coaching methods chosen as a prerequisite for being considered for this review. However, only one of the studies used the term “life coaching” [25], while the other studies described the coaching as integrative health coaching [26], wellness coaching [27], or co-active coaching [7]. One study did not classify the coaching method used [23,24].

All studies included a description of how the coach guided the patients to setting their own goals by using different systematic methods such as specific, measurable, achievable, realistic, and time-scaled (SMART) objectives [25], or the wheel of life [7].

#### Education of coaches

In three of the studies, the coaching was conducted by health professionals trained in coaching but without any certified coach training [23-26]. The other two projects used professional certified coaches [7,27].

### Patient outcome

The impact of the coaching on objective health outcomes was investigated in two studies. In one of the randomized controlled trials (RCTs), no significant difference existed between the improvements in the intervention group and the control group; however, in a subgroup of patients with a high baseline HbA1c, significant improvements were found [26]. In a pre-post design in the case study, HbA1c decreased significantly in 6 of 9 poorly controlled adolescents after having participated in the coaching program.

Improvement in the quantitative data reported by the patients such as stress, adherence to medications, perceived health status, high goal attainment, quality of life, and decreased depression and anxiety was disclosed in three of the studies [25-27]. However, the other RCT did not show any improvement in psychological adjustment to illness and health-related quality of life after the intervention, whereas the self-efficacy in the intervention group increased significantly during the follow-up period [23].

The qualitative data from the interview with the patients showed that the patients were very satisfied with the coaching intervention and felt encouraged to a) adopt a new view of themselves and their illness, b) try out new methods, and c) work systematically with their goals [7,24,25].

### Methodological quality

Assessment of the methodological quality is illustrated in Table 2. The quality scores range from 0–7 points (0-100%) as ‘done’ gave 1 point and ‘not done’ or ‘not clear’ gave 0 point. Two RCTs met all of the quality criteria [23-26]. Schneider et al. [25] did not receive any points because the description of the methodology was unclear.

**Table 2:** Criteria list inspired by LP Moja [21], JM Olsen [2], and Cherafhi-Sohi S et al. [22]

**Study**	**Random assignment**	**Use of control group**	**Baseline comparability**	**Follow-up**	**Analysis of data clearly reported**	**Used validated outcome measure/ instruments**	**Description of the intervention (purpose, frequency, length, coaching elements, coach training)**
Schneider et al.	Not clear	Not done	Done	Not Done	Not done	Not done	Not clear
Izumi et al./Hayashi et al.	Done	Done	Done	Done	Done	Done	Done
Wolever et al.	Done	Done	Done	Done	Done	Done	Done
Galantino et al.	Not clear	Not done	Done	Done	Done	Done	Done
Ammentorp et al.	Not done	Not done	NA*	Done	Done	Done	Done

‘Done’: 1 point, ‘Not done’ or ‘Not clear’ 0 points.

*N/A implies not applicable based on qualitative methodology used.

## Discussion

Unfortunately, only a limited number of trials were identified as using a life coaching process reporting health outcomes, and as the quality of the studies varied widely, it was not possible to draw definite conclusions. Therefore, this review can only present tendencies for patient outcomes and a preliminary description of an effective life coaching intervention.

The two studies investigating objective health outcomes (HbA1c) showed mixed, but promising results [7,26]. In the case study, it was possible to obtain significant improvements in a patient group that usually does not benefit from intensified interventions [7] and in the randomized trial; the patients with an elevated baseline HbA1c significantly improved their metabolic control [26].

The fact that it was a group of disadvantaged patients that showed significant improvement may indicate that some patients can benefit from being met with another approach and a different type of communication. Results from research in understanding and developing resilience to chronic diseases may explain the findings. Resilience is a psychological construct referring to an individual’s capacity to maintain psychological and physical well-being in the face of adversity, including physical illness [28,29]. Some of the characteristics associated with resilience in physical illness are self-efficacy, self-empowerment, acceptance of illness, determination, optimism, hope, and mastery [29]. Therefore, patients with low self-efficacy and self-empowerment may benefit from life coaching as it is based on methods aimed to improve these characteristics [28,29].

One of the quantitative studies evaluating the outcomes reported by the patients did not find any significant improvements [23] while two other studies showed significant improvements [7,26]. These results together with findings in the qualitative studies [7,24] supported the tendencies described above by eliciting improved goal attainment, self-reported adherence, and improved health status and self-esteem.

In the review, two studies differed from the others by using professional certified coaches from outside the health care system. These studies included only 20 and 9 patients respectively [7,27], but based on the findings in the pilot project [7] and on the literature, pointing out the difficulty for providers to change their consulting style [30], using external professional certified coaches appears promising.

We did not distinguish between face-to-face coaching and telephone coaching, because the principles and dynamics are regarded as being very similar, and telephone coaching is used as a logistically simpler way of obtaining the same results [31]. Unfortunately, it has not been possible to find any studies comparing the outcome of the different methods.

The main challenge in conducting this review turned out to be the selection of life coach interventions. Although we tried to define the intervention as precisely as possible, there were studies in which it was difficult to distinguish between health coaching and life coaching [32]. However, we decided to maintain this distinction; firstly because health coaching includes a very broad spectrum of studies describing different methods from education or instruction related to specific situations [33] to several more or less structured coaching interventions aimed at reaching specific values such as a specific blood pressure value [34]. Secondly, many of the studies described as health coaching have used a method very similar to the method described in motivational interviewing studies [35].

If we had chosen all coaching interventions, including health coaching interventions, the problem with distinguishing coaching from motivational interviews and more instructive coaching interventions would probably have diminished the value of this review. This assumption is supported by the research in health coaching interventions [2,20,36].

Although the included studies were based on the definition of life coaching, only one of the studies referred to the coaching method as life coaching; however, the description of the method used showed that the coach addressed the entire life of the client and as the agenda was decided by the client and not by the coach [37], this corresponded to the definition of life coaching [12,38].

The topic of the review and the problems described above made it challenging to ensure that the literature review became sufficiently comprehensive; however, by being very systematic and by following the recommendations in the literature [21,39,40], we feel reasonably confident that we have identified all relevant studies.

## Conclusions

This systematic review has shown that the experiences of using life coaching as a supplement to the medical approach in health care are very limited and the quality of the data from the few studies described are of varied quality. Notably, the review has pointed to some interesting aspects which make it relevant to undertake further research into how this method can improve the well-being of patients. In order to optimize reviews like this, it is very important that the description and categorization of the coaching methods are described more comprehensively, although this can only be done to a certain degree. In order to get a closer look at what is in the ‘black box,’ we suggest that research into this area is supplemented by a more qualitative approach investigating the content, the communication process, and the interaction.

## Competing interests

The authors declare that they have no competing interests.

## Authors’ contributions

JA and PEK developed the idea for the study. JA, PEK, LU, and EBC revised the search strategy and participated in reviewing the identified articles. JA wrote the first draft of the paper. All authors participated in the interpretation of the data; they contributed to the critical revision of the paper and approved the final version of the manuscript.

## Pre-publication history

The pre-publication history for this paper can be accessed here:

http://www.biomedcentral.com/1472-6963/13/428/prepub
